# Client satisfaction to methadone maintenance treatment program in Myanmar

**DOI:** 10.1186/s13011-021-00429-z

**Published:** 2022-01-03

**Authors:** Sun Tun, Balasingam Vicknasingam, Darshan Singh, Nyunt Wai

**Affiliations:** 1 Myanmar Medical Association, Yangon, Myanmar; 2 Centre for Drug Research, Universiti Sains Malaysia, Penang, Malaysia

**Keywords:** Methadone, Verona service satisfaction scale for methadone treatment – VSSS-MT, Treatment satisfaction, Opiate, Myanmar

## Abstract

**Background:**

To tackle the long-standing opioid misuse problem, Myanmar introduced the methadone maintenance treatment (MMT) program in 2006, starting with 260 clients. Since then, the program has been expanded across different geographical sites in the country. This study was done in 2017 to explore the treatment satisfaction of the clients towards the MMT program.

**Method:**

A total of 210 clients with a minimum of six-month treatment history enrolled in five MMT program sites across Myanmar were recruited through stratified random sampling. Administering the Verona Service Satisfaction Scale for Methadone-Treatment (VSSS-MT), this study assessed the satisfactory responses under three categories viz., 1) clinic staff professional skills; 2) basic drug intervention; 3) specific intervention (individual rehabilitation and psychotherapy).

**Results:**

The majority (89%, *n* = 186) of the respondents were satisfied with the current MMT program. Specifically, 89.5% (*n* = 187) were satisfied with the clinic staff professional skills category, 91.9% (*n* = 192) with the basic program intervention and 74.6% (*n* = 156) with specific interventions. Among the respondents, treatment satisfaction with the MMT program was higher (*p* < 0.05) in those (i) with a higher quality of life score in physical, psychological, social and environmental categories, ii) who were satisfied with their current marital and leisure status, and those iii) who consumed alcohol. Results from stepwise binary logistic regression showed alcohol consumption and physical health status had a significant association with MMT treatment satisfaction.

**Conclusion:**

Treatment satisfaction of the clients, in general is high. However, the lower percentage of satisfied clients (74.6%) for the specific interventions category compared with 89.5 and 91.9% respectively for staff and basic drug management categories highlights the need for improvement in this category for overall enhancement of the MMT program.

## Background

The Global Health Observatory data from the World Health Organization (WHO) had highlighted that in 2014, 45% of the 147 responding countries had methadone therapy chiefly for OUD detoxification purposes [28]. The World Drug Report 2020 from the United Nations Office on Drugs and Crime (UNODC) estimated that 57.8 million people had used opioids in 2018, and 80% have lost 42 million years of “healthy life” to OUD [26]. Drug dependence has been described as a multi-factorial health disorder with a high possibility for relapse even after seeking treatment. Thus, it is imperative that people who use opioids be enrolled in opioid substitution treatment programs to cut the vicious cycle of addiction and halt the spread of infectious diseases such as HIV.

Myanmar, a country that accounted for 7% of the world’s opium production [26], continued to face significant problems with opioid misuse. To curb this menace, punitive laws have been enforced, where people who use drugs can be detained and jailed for misusing illicit substances. Given the opiate misuse threat and its link with HIV spread, the Methadone Maintenance Treatment (MMT) program was first introduced in Myanmar in 2006 as a harm reduction intervention. Owing to its success, the MMT program has been expanded throughout Myanmar in line with the recommendations from the National Drug Control Policy [3]. The DDTRU first started the MMT program in Myanmar with 260 clients before it was gradually upscaled [6]. As of 2020, 26,016 people who inject drugs (PWIDs) (i.e. 28% of 93,000 estimated PWIDs in Myanmar) were taking methadone [21]. Like any other program, this harm reduction intervention could have faced challenges, obstacles and limitations in its service delivery. It would be beneficial to monitor such impediments by assessing client satisfaction towards the MMT program.

It has been pharmacologically shown that the MMT program is effective as it is associated with decreased opioid use [29]. Improvement in the quality of life (QoL) has been reported for clients enrolled in the MMT program for the short term [8], as well as long-term duration [4]. Furthermore, the MMT program clients were less likely to resort to illicit opioid use [30] and were reported to be satisfied with ancillary treatment services often provided in the MMT program [9]. Given the rapid expansion of the MMT program, several validated tools have been developed to assess clients’ treatment satisfaction with the MMT program. Commonly used tools include the Client Satisfaction Questionnaire (CSQ-8) [11], the Service Satisfaction Scale (SSS-30) [19] and the Verona Service Satisfaction Scale (VSSS-32) [18]. Satisfaction with HIV/AIDS Treatment Interview Scale (SATIS) is a tool that could be modified and used to assess satisfaction towards the methadone services.

A recent situational analysis indicated that MMT programs are bound to have several limitations or barriers. They are inconvenient operational hours, long travel distance to MMT sites, lengthy registration process, coping with the continuous drug use issue among clients, long induction period, and a lack of confidentiality [14]. In view of ongoing MMT program expansion in Myanmar, it has become crucial to explore clients’ satisfaction towards the MMT program and its ancillary services so that timely remedial measures could be taken to enhance the user-friendliness of the program. As such, this study aims to evaluate the satisfaction of the clients enrolled in Methadone Maintenance Treatment Program in Myanmar with the following objectives:

1) to explore clients’ overall satisfaction towards the program in general;

2) to assess clients’ satisfaction towards a) professional skills of the clinic staff; b) basic drug intervention (Methadone administration); c) specific intervention such as individual rehabilitation and psychotherapy,

3) to find out if any of clients’ characteristics are associated with their satisfactory response.

## Materials and methods

### Study design, respondents and location

Two hundred and ten clients enrolled in the MMT program across different geographical provinces in Myanmar were recruited for this study. We used the stratified random sampling approach to recruit a quota sample of 42 respondents from each of the following locations across Myanmar; Yangon Region, Mandalay Region, Lashio (from Shan State), Kawlin (from Sagaing Region), and Mohnyin (from Kachin State). Surveys were conducted through face-to-face interviews by trained researchers at the targeted MMT facilities. Respondents were assured that this study would not collect their personally identifiable information and all the study data would be kept confidential. Each interview session lasted 25 to 30 min. All the respondents gave informed consent and were compensated for their time.

### Inclusion and exclusion criteria

The inclusion criteria for the study were as follows; a) Age 18 years and above, b) self-reported as a methadone client currently enrolled in a formal MMT program in Myanmar, and c) at least a minimum of six-month methadone treatment history. We believe six-month treatment history would be long enough to provide us with a better picture of the client’s satisfaction with the MMT program and ancillary services.

Those observed to be in an incoherent state of mind and those who were reluctant to participate voluntarily were excluded from the study.

There were full cooperation and participation from the methadone clients who were selected for participation in the survey. Drop-in-centres managers and methadone clinic doctors explained to the client the initial information session before the survey. During the interview visit, the research team also explained the importance of this survey and the potential benefit of the survey results in the operation of the methadone programme in different areas.

### Measures

We collected the study data from May 2017 to July 2017. A semi-structured questionnaire was used to elicit respondents’ socio-demographic characteristics such as current age, gender, employment status, MMT treatment history, HIV status, illicit drug use history, and service satisfaction with the MMT program. We also used the Verona Service Satisfaction Scale for Methadone Treatment (VSSS-MT) [5], the Addiction Severity Index- Lite (ASI) scale [13], and Quality of Life Scale developed by the World Health Organization (WHOQOL-BREF) [27]. This study also used The Timeline Follow Back (TLFB survey) [15] tool to determine respondents’ substance use history in the last 7 days. The responses were coded with a Likert five-point scale (1 denotes the worst satisfaction and 5 vice-versa). We reported satisfaction with methadone service (VSSS-MT score) based on the 27 VSSS-MT questions. Those rated above the 3 out of 5 Likert scales of each questionnaire were summed for the total satisfaction score. Over 80 and above was regarded as “satisfied”, and the rest were “not satisfied” on that dichotomous output of satisfaction. The satisfactory responses were grouped into 3 categories: 1) professional skills of clinic staff (doctor, nurse, counsellor; 2) basic drug intervention (e.g., instruction between visits, side-effects), and 3) specific intervention (e.g., individual rehabilitation and psychotherapy, group therapy). Two trained bilingual translators translated the survey questionnaires and validated scales from English to Myanmar (Burmese).

### Urinalysis

All the respondents were required to undergo a supervised urine drug test for the following substances; Methadone, morphine, cannabis, methamphetamine, amphetamine, and benzodiazepine. All the urine specimens were appropriately discarded at the end of the interviews to avoid legal consequences.

### Statistical analysis

We analysed all the study data with Stata 14.0 software. First, to identify the association between VSSS-MT Treatment satisfaction and categorical variables of patient characteristics, a Chi-squared test was performed for two tailed-test at a significance level of *p* < 0.05. Next, we compared the mean scores of certain parameters (VSSS-MT scores) of interest with a *t*-test. We ran the Binary logistic regression for identifying the predictors to the outcome “VSSS-MT score” at *p* < 0.05 with confounding variables controlled. The Cox regression model for HIV infection was used to explore the relationship between treatment satisfaction and HIV status.

### Ethical measures

The study had been approved by the Human Ethics and Research Committee of Universiti Sains Malaysia and then, Myanmar Ministry of Health and Sports, Department of Medical Research (No: Ethics/DMR/2017/057) [17].

## Results

### Demographic characteristics

In this study, the majority (98.6%) of clients (*n* = 210) were males and only 1.4% were females. The mean age was 33.3 years (SD = 8.85, range: 20–76 years). All clients took their daily methadone supply from MMT clinics, and 83% (*n* = 173/210) were in their first-time treatment of MMT. The average daily methadone dose was 83 mg (SD = 53, range: 20 mg–300 mg/ day), and the average duration was 28 months (SD = 28.5, range: 6–127 months). Almost two thirds (63%, *n* = 132) received ≤80 mg/day dosage, while 76 (37%) received a higher dosage of more than 80 mg/ day. The average Body Mass Index (BMI) was 20.5 (SD = 3.4, range: 14.0–33.3). Regarding education status, 75.6%, (*n* = 159/ 210) had primary to high school education, 21.43% had a college education, and only six clients lacked formal education. Almost half of the respondents (46.4%) were single or divorced, while 40.58% were married.

As for the employment status in the last 3 years, most respondents (93.43%) were employed, and only 8.57% were unemployed. Twenty-nine respondents worked as outreach workers or peer educators for people with substance use problems.

### Variations in treatment satisfaction rating scores

Treatment satisfaction rating scores varied from study site to site, with scores with scores being lower in bigger cities (Yangon, Mandalay) and higher in smaller cities (Kawlin, Lashio and Mohnyin). For clients who rated 3 and above on the Likert scale of 5 for all items in the questionnaire, the mean score for satisfaction with methadone service (VSSS-MT scale) was set as 100 (range: 58–131), while the score over 80 was designated as “much satisfaction”. While 88.6% (*n* = 186) of clients were highly satisfied with their MMT program, there were variations in the ratings among the three categories: professional skill items (doctor, nurse, counsellor and worker) was rated 3.89 out of 5, basic intervention items, 3.83 and specific intervention items 3.42. This highlights that among three categories of methadone service that captured treatment satisfaction, specific intervention category scored the lowest.

The VSSS-MT score was mentioned based on responses to each question, as shown in Table 1.

**Table 1:** Table shows VSSS-MT Treatment satisfaction score among respondents

Respondent characters	Sub groups	Number (n)	Total VSSS-MT score (SD)	*p* value
**Methadone dose categories**	less than or equal 80 mg more than 80 mg	132 76	98.61 (13.97) 103.11 (14.28)	0.0276**
**Methadone duration**	less than or equal 2.4 years more than 2.4 years	120 89	100.83 (14.40) 99.40 (13.93)	0.4755
**Methadone treat time**	First time treatment More than first time	173 35	101.34 (13.95) 94.74 (14.46)	0.0120**
• less than or equal 80 mg	First time treatment More than first time	109 22	100.03 (13.45) 91.54 (14.96)	0.0091**
• more than 80 mg	First time treatment More than first time	63 13	103.71 (14.67) 100.15 (12.27)	0.4167
• less than or equal 2.4 years	First time treatment More than first time	95 24	102.68 (13.52) 93.54 (15.98)	0.0052
• more than 2.4 years	First time treatment More than first time	78 11	99.69 (14.37) 97.36 (10.61)	0.6067
HIV status (HIV)	Not infected Infected	126 74	99.23 (13.84) 102.35 (14.81)	0.1351
Hepatitis C status (HCV)	Not infected Infected	77 71	103.21 (13.35) 98.66 (14.03)	0.0452**
Hepatitis B status (HBV)	Not infected Infected	166 15	101.14 (14.04) 101.73 (12.19)	0.8754
Tuberculosis (TB) treatment history	Not treated Treated	147 54	100.92 (13.71) 100.65 (14.00)	0.9021
Sexually Transmitted infection (STI) history	Not infected Infected	164 45	100.06 (13.69) 100.80 (16.01)	0.7577
Age	Younger and equal 35 years Older than 35 years	128 81	100.82 (14.43) 99.27 (13.82)	0.4433
Body Mass Index (BMI)	Less than mean BMI (20.5) More than mean BMI	122 84	100.33 (14.87) 100.48 (13.06)	0.9412
Currently on antiretroviral therapy (ART)	No On treatment	141 68	99.08 (13.53) 102.59 (15.29)	0.0938
Education	Up to primary More than primary	48 161	104.77 (15.35) 98.86 (13.58)	0.0110**
Recent work	Unemployed Employed	24 182	96.95 (15.41) 100.93 (13.81)	0.1926
Current Peer/ Outreach	No Peer/outreach	180 29	100.52 (14.27) 98.38 (13.77)	0.4528
ASI for Employment	Low score High score	102 104	99.39 (13.39) 100.83 (15.06)	0.4711
ASI for Alcohol Use	Low score High score	28 36	105.96 (10.96) 102.22 (14.48)	0.2600
ASI for Drug Use	Low score High score	162 47	100.96 (14.21) 97.66 (13.97)	0.1604
ASI for Legal Status	Low score High score	14 14	101.64 (15.57) 94.29 (10.86)	0.1590
ASI for Family/ Social Status	Low score High score	139 70	100.90 (13.71) 98.87 (15.09)	0.3306
Marital status	Currently married Single/separated	84 122	98.62 (14.51) 101.48 (14.01)	0.1568
Income	Lower (than average) Higher	131 77	99.65 (14.97) 101.18 (12.89)	0.4542
Current marital status satisfaction	Not satisfied Satisfied	20 189	96.65 (19.01) 100.60 (13.59)	0.2375
**WHO Quality of life (QOL) total score**	Low High	88 121	93.63 (14.42) 105.02 (11.96)	0.0001***
Physical QOL score	Low High	53 156	88.94 (15.06) 104.05 (11.65)	0.0001***
Psychological QOL score	Low High	41 168	91.07 (15.32) 102.45 (12.99)	0.0001***
Social QOL score	Low High	71 138	94.86 (15.47) 102.98 (12.68)	0.0001***
Environmental QOL score	Low High	54 155	90.81 (14.83) 103.50 (12.42)	0.0001***
Current leisure status satisfaction	Not satisfied Satisfied	29 180	92.38 (16.59) 101.48 (13.39)	0.0012**
• Current leisure status with family	Not satisfied Satisfied	119 90	100.03 (14.27) 100.48 (14.15)	0.8200
• Current leisure status with friend	Not satisfied Satisfied	135 74	99.52 (14.76) 101.50 (13.08)	0.3354
• Current leisure status alone	Not satisfied Satisfied	159 50	101.09 (13.71) 97.44 (15.42)	0.1123
**Abuse encountered within 30 days**				
• Psychological abuse	Not experienced Experienced	183 22	100.01 (13.91) 101.05 (16.64)	0.7474
• Physical abuse	Not experienced Experienced	203 1	100.10 (14.26) 102.00 (.)	.
• Sexual abuse	Not experienced Experienced	204 1	100.07 (14.20) 111.00 (.)	.
**Urine illicit substance findings**				
• Urine Morphine	Absent Present	93 116	100.62 (14.13) 99.90 (14.29)	0.7137
• Urine Tetrahydrocannabinol (THC)	Absent Present	185 24	100.42 (14.40) 98.71 (12.59)	0.5802
• Urine Methamphetamine	Absent Present	158 51	100.93 (14.62) 98.02 (12.65)	0.2034
• Urine Amphetamine	Absent Present	191 18	100.19 (14.50) 100.56 (10.55)	0.9167
• Urine Benzodiazepine	Absent Present	137 72	100.84 (14.95) 99.04 (12.62)	0.3853
Last heroin injection within 30 days	No Yes	93 115	102.35 (14.15) 98.44 (14.08)	0.0483**
Frequency of injection	No or few injections (mean = 7) Higher	96 112	102.46 (13.64) 98.35 (14.48)	0.0373**
Needle sharing within 30 days	Not shared Shared	99 4	99.21 (15.26) 102.00 (1.41)	0.7169
Life time sharing of needle and syringes	Not shared Shared	106 102	98.72 (12.66) 101.58 (15.47)	0.1452

Independent t-test *p* value: ** significance < 0.05, *** significance < 0.001

Table explanation: This table explains the result of the significant difference of the total score of the VSSS-MT questionnaire with the respondent characteristics

### Differences in VSSS-MT scores among various characteristics of the respondents

We also analysed whether there were significant differences in the MMT satisfaction score with different types of client behaviours and reported characteristics (See Table 2).

**Table 2:** Table shows total VSSS-MT score differences by the characteristics of patients

Variable	Adjusted Odds Ratio (aOR) (95% CI)	***p*** value
Alcohol	5.57 (1.2, 25.88)	0.029**
Physical quality of life (QOL) score	11.87 (4.29, 32.84)	0.000***

Stepwise binary logistic regression, *p* value: **significance *p* < 0.05, ***significance *p* < 0.001.

**Fig. 1:**
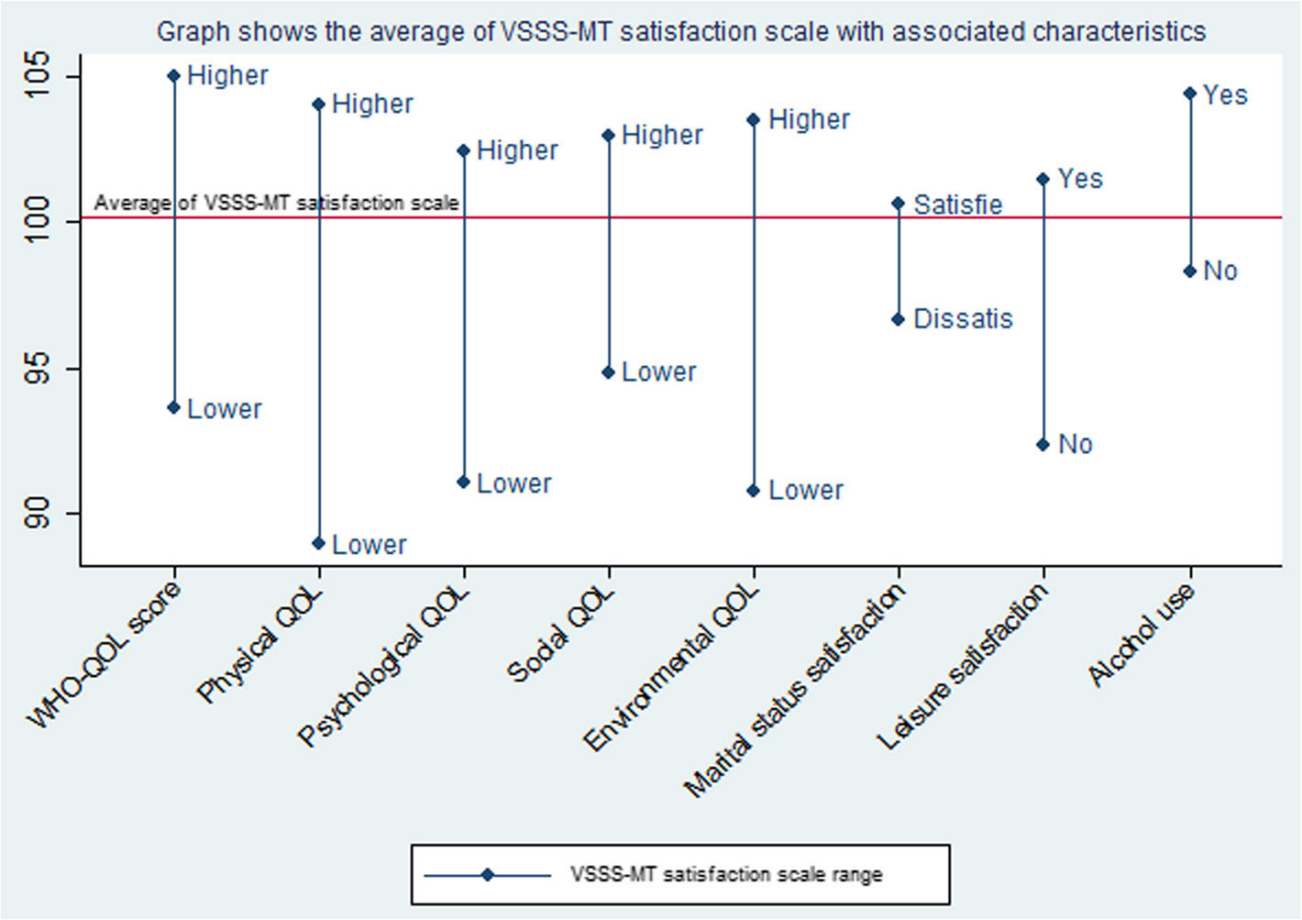
Graph shows the average of VSSS-MT satisfaction score (scale) with associated characteristics. Legend: Y-axis shows the average result of the VSSS-MT total score

Among the respondents, those with a higher quality of life score on physical, psychological, social and environmental categories, those who were satisfied with their current marital and leisure status, and those who consumed alcohol had higher MMT treatment satisfaction scores of over 80 (*p* < 0.05) (See Fig. 1.). Those on high methadone dose (> 80 mg/day) also had higher MMT satisfaction scores compared with clients who received the lower dose (≤80 mg/day) (*p* = 0. 0276).

### Stepwise regression analysis to identify factors predicting the methadone service satisfaction

To identify the predictors that were linked with the primary outcome dependent variable of MMT satisfaction, characteristics that were significantly associated with one another (*p* < 0.05) were considered in the regression analysis. The backward stepwise binary logistic regression analysis was done among the significant parameters in the model to identify final predictors to the outcome group with MMT satisfaction (See Table 3).

**Table 3:** Table shows correlates of VSSS-MT score from stepwise

Variable	Frequency (n and %)	Mean (SD) VSSS-MT score^a^	Range/ Categories
Verona Service Satisfaction Scale for Methadone-Treatment (VSSS-MT) score (total)	209 (100%)	100.22 (14.19)	58–131
Not much satisfied	23 (11%)		
Much satisfied	186 (89%)		
**VSSS Item Categories**			
Professional Skills Items	209	3.89 (0.64)	1.5–5
Basic Interventions Items	209	3.83 (0.54)	2.3–5
Specific Interventions Items	209	3.42 (0.68)	1.5–4.9
**VSSS Items Description**			
1. Helping patient deal with problem	209	3.77 (0.96)	Basic skill items
2. Doctors’ ability to listen	208	3.97 (0.83)	Professional Skills Items
3. Psychologists’ ability to listen	206	3.99 (0.86)	Professional Skills Items
4. Doctors’ manner	208	3.92 (0.84)	Professional Skills Items
5. Psychologists’ manner	204	4.02 (0.77)	Professional Skills Items
6. Referring to other specialists	201	3.76 (0.87)	Basic skill items
7. Overall satisfaction	208	4.22 (0.76)	Basic skill items
8. Nurses’ manner	209	3.91 (0.84)	Professional Skills Items
9. Social workers’ manner	205	3.73 (1.00)	Professional Skills Items
10. Improving relationship between patient and relatives	208	4.00 (0.84)	Basic skill items
11. Helping family members to understand patient’s problems	209	4.01 (0.81)	Basic skill items
12. Nurses’ knowledge of patient’s medical history	209	3.78 (0.94)	Basic skill items
13. Information on addiction	209	3.72 (0.93)	Basic skill items
14. Helping patient in relationships outside the family	209	3.44 (1.04)	Basic skill items
15. Instructions between visits	209	3.79 (0.85)	Basic skill items
16. Helping patient to look after himself	209	3.98 (0.77)	Basic skill items
17. Nurses’ ability to listen	208	3.83 (0.84)	Professional Skills Items
18. Social workers’ ability to listen	209	3.75 (0.92)	Professional Skills Items
19. Help received for methadone side effects	207	3.88 (0.85)	Basic skill items
20. Individual rehabilitation	209	3.64 (1.07)	Specific Interventions Items
21. Individual psychotherapy	209	3.67 (1.05)	Specific Interventions Items
22. Family therapy	208	4.19 (0.81)	Specific Interventions Items
23. Activities organised by centre	209	3.82 (0.97)	Specific Interventions Items
24. Group psychotherapy	209	3.33 (1.06)	Specific Interventions Items
25. Sheltered work	208	3.03 (1.23)	Specific Interventions Items
26. Help by the centre at home	204	2.51 (1.27)	Specific Interventions Items
27. Help to join in activities separate from the centre	209	3.16 (1.17)	Specific Interventions Items

^a^Table explanation: This table explains the result of the mean score for each components of the VSSS-MT questionnaire set

After adjusting the potential confounding variables, this analysis estimated the association between the independent variables and the outcome variable of the service satisfaction score category. Those who used alcohol were 6 times (aOR 5.57, 95%CI; 1.20–25.88, *p* = 0.029), and those who had good physical health (higher score in physical quality of life) were 12 times (aOR 11.87, 95%CI; 4.29–32.84, *p* = 0.000) more satisfied with their MMT program. When checking for the multi collinearity, mean variance inflation factor (vif) was 1.61 and none of the variables had more than 10. The regression model alpha ratio was set at 0.05.

### Reported infection status of the patients

About one-third (36.5%, *n* = 76) of respondents were on high-dose methadone (more than 80 mg/ day) and the rest 63.5% (*n* = 132) on low-dose methadone (less than 80 mg/ day). Almost half (47%, *n* = 71/ 148), of the respondents reported hepatitis C virus infection (HCV), 37% (*n* = 74/200) reported Human Immunodeficiency Virus (HIV) infection, while 16% (*n* = 34) reported HIV and HCV co-infection. Forty-five respondents (21.53%) reported sexually transmitted infections, and 15 respondents (8.29%) reported Hepatitis B infection. Out of reported HIV respondents, 68 (92%) were on antiretroviral therapy (ART) with an average ART duration of 30 months (SD = 33, range: 1–132 months). Taking high methadone dose (more than 80 mg) was found to have a significant association with having antiretroviral therapy (*p* = 0.039) [24].

### Estimation of treatment satisfaction by different infection statuses

We also ran a univariate cox proportional hazard model analysis to estimate treatment satisfaction based on the acquired data. In this survival analysis, which is similar to that of [10], “the methadone dose” was set as a “time” variable, and “Treatment satisfaction” was set as a “failure event”. We then analysed the HIV status and HIV and HCV co-infection status against different methadone doses to estimate and compare incidence rates on their treatment satisfaction (*p* < 0.05).

The results show that, after controlling the methadone dose variable (IRR = 0.49, *p* = 0.000), treatment satisfaction was 2 times higher among HIV negative patients compared with HIV positive patients, and the satisfaction was 1.7 times higher among non-co-infected patients compared with co-infected patients (IRR = 0.59, *p* = 0.000). The higher rate of satisfaction incidence among HIV negative respondents was 1.49% and among HIV positive respondents was 0.72% after adjusting the methadone dose (p = 0.000). A higher dose was associated with increased satisfaction scores (*p* = 0.037) in HIV negative respondents [25].

Higher satisfaction incidence was 1.2% among non-co-infected patients and 0.71% among co-infected patients after adjusting for doses. In other words, co-infected patients were less likely to be satisfied with the program than non-co-infected patients (*p* < 0.001). This Cox regression, in which univariate analysis was done for the interested variable “Treatment satisfaction”, and this analysis did not take into account other confounding variables. Despite the limitation of cross-sectional study type and potential bias, the estimation of the incidence rate of “treatment satisfaction” was analysed with a similar way of analysis [7].

## Discussion

This study found that 88.6% (*n* = 186) of clients were highly satisfied with the MMT program implemented in their respective localities in Myanmar.

Among three categories of methadone service that captured treatment satisfaction, the rating for professional skill items (skills of doctor, nurse, counsellor and workers) scored the highest (3.89 out of 5), while items in specific intervention category scored the lowest. A Vietnamese study [22] also reported high satisfaction in professional skill items. It was reported that MMT programs that offered ancillary services as specific intervention improved MMT satisfaction ([12, 22]. It was reported that limitations or barriers such as dosing spaces, waiting areas and staff shortage could affect treatment satisfaction [2]. Thus the specific intervention component in MMT programs could be enhanced by providing ancillary services such as counselling, medical services (Individual rehabilitation), psycho-social services (psychotherapy and group therapy), and psychiatric care. Thus, the specific intervention component in MMT programs could be enhanced by providing ancillary services such as counselling, medical services (Individual rehabilitation), psycho-social services (psychotherapy and group therapy), and psychiatric care. As the specific intervention focuses on individual and group counselling, family support, and the centre supports at home, the methadone intervention can be more effective if integrated with individual and/or group counselling, employment or family services [1]. Moving in this direction could improve clients’ treatment adherence and satisfaction.

Regarding the program categories, the ratings for satisfaction in this study were higher than those reported by a study from Spain (3.83 vs 3.5 for basic intervention items and 3.42 vs 3.1 for specific intervention) [23]. People in Myanmar are known for their friendliness, easy-going lifestyle and a high tolerance for life’s inconveniences. This might be a factor for the high satisfaction scores observed in this study.

Many factors influence MMT treatment satisfaction. These include recent heroin injections, use of benzodiazepines, HCV infection, high addiction severity index (ASI), alcohol consumption and family/ social status. Limitations or barriers in methadone treatment such as dosing spaces, waiting areas and staff shortage [2], methadone dispensing hour, methadone dose change, the number of patients per centre, frequency of information about methadone dose changes, and lower social dysfunction subscale of General Health Questionnaire-28 [23]; could also affect treatment satisfaction.

Our stepwise binary logistic regression result showed alcohol consumption and physical health were significantly associated with methadone treatment satisfaction. Although Myanmar is a majority Buddhist country, alcohol consumption is common among its people.

There were a few limitations in this study. First, respondents for this study were randomly recruited from a few MMT clinics. Hence there could be bias by not enrolling clients unsatisfied with their MMT program. There is a limitation to make generalisations from the results bias the data were based on one-time self-reporting. The result’s further potential bias could be contributed by the selection criteria of a minimum 6-month treatment. The National indicator from the DDTRU (Drug Dependency Treatment and Research Unit) specified the client retention in the methadone treatment as “those clients who were taking methadone at least 6-month period”, and this survey excluded the satisfactory situation of the clients who were less than 6 months of the treatment. The 2018 annual data [16] showed 70% received at least 6 months, and we could interpret that the survey results were derived from the samples representing the majority of the clients. As the respondents were patients currently taking methadone, they might be reluctant to take risks in expressing negative views; Hawthorn’s effect thus could not be ruled out despite our efforts in ensuring confidentiality. We analysed this study with the Chi-squared test repeatedly to identify the satisfaction of the methadone clients. So, there could be a potential increase in type I error as there were multiple comparisons to test the null hypotheses [20]. Finally, factors like early drop-outs and refusal to participate in the study, could be a threat to the internal validity and generalizability of the results. In conclusion, our results indicated that clients in the MMT program in Myanmar were highly satisfied with their methadone treatment program. However, the lower percentage of satisfied clients for the specific intervention category compared with staff and basic drug management categories highlights the need for improvement in the Specific intervention component in MMT programs by providing ancillary services such as counselling, medical services (Individual rehabilitation), psycho-social services (psychotherapy, and group therapy), and psychiatric care.

## Data Availability

The [.dta] data used of the findings in this study are available from the Centre for Drug Research upon the approval.

## Abbreviations

aOR: Adjusted Odds Ratio
ART: Antiretroviral therapy
ASI: Addiction Severity Index
BMI: Body Mass Index
DDTRU: Drug Dependency Treatment and Research Unit
HCV: Hepatitis C Virus
HIV: Human Immunodeficiency Virus
IRR: Incidence Rate Ratio
MMT: Methadone Maintenance Treatment
PWID: People Who Inject Drugs
SD: Standard Deviation
VSSS-MT: The Verona Service Satisfaction Scale for Methadone-Treatment
WHO: World Health Organization
WHOQOL-BREF: generic Quality of Life Scale developed through the World Health Organization
