# Correction to: Client satisfaction to methadone maintenance treatment program in Myanmar

**DOI:** 10.1186/s13011-022-00432-y

**Published:** 2022-02-02

**Authors:** Sun Tun, Balasingam Vicknasingam, Darshan Singh, Nyunt Wai

**Affiliations:** 1 Myanmar Medical Association, Yangon, Myanmar; 2 Centre for Drug Research, Universiti Sains Malaysia, Penang, Malaysia

**Correction to: Subst Abuse Treat Prev Policy 17(1):2 (2022)**

**https://doi.org/10.1186/s13011-021-00429-z**

Following publication of the original article [1], the authors identified an error in the table captions. The captions of Tables 1, 2 and 3 were swapped. The original article has been updated with the correct captions.
